# Green workspace and urban health: exploring the impacts of industrial robotics in pollution emissions and public health

**DOI:** 10.3389/fpubh.2024.1445746

**Published:** 2024-08-02

**Authors:** Shule Yu, Minghan Hu, Jiancheng Li, Xueling Yan

**Affiliations:** ^1^School of Economics, Fudan University, Shanghai, China; ^2^School of Economics, Sichuan University, Chengdu, China

**Keywords:** green workplace, pollution reduction, industrial robotics, environmental-friendly production, public health

## Abstract

**Introduction:**

This study addresses a critical gap in understanding how technological advancements, specifically industrial robots, influence urban pollution emissions and public health. The rapid evolution of technology and changing working conditions significantly affect these areas, yet research has not extensively explored this domain.

**Methods:**

Utilizing 2018 China Labor-force Dynamic Survey (CLDS) dataset, this study examines the impact of industrial robots on public health. An analytical framework is employed to assess the correlation between the adoption of eco-friendly industrial robots and improvements in worker health, attributed to the reduction of pollution emissions.

**Results:**

The findings reveal that the adoption of industrial robots significantly enhance both public physical and mental health. This study also identifies potential demographic heterogeneity in the effects of industrial robots. The benefits are more pronounced among non-insured manual female workers who are older, have lower education levels, and hold rural hukou. These benefits are closely linked to improvements in the quality of the production environment and reductions in pollution emissions at both macro and micro levels.

**Discussion:**

The study underscores the significant potential of industrial robots to positively impact urban health, advocating for strategies that promote the development of safer, greener environments.

## 1 Introduction

In recent years, urbanization in developing countries has rapidly accelerated due to the significant growth of the global economy. Currently, over 50% of the global population lives in urban areas-this is 3.9 billion, and by 2030, this number will rise to about 5 billion (1). Urbanization has a positive impact on residents’ quality of life by enhancing the quality of medical services and infrastructure available to them (2). However, urbanization also leads to the high concentration of secondary industries, such as manufacturing, within limited spaces (3). This intensive production results in severe environmental pollution, contributing significantly to overall urban pollution. For example, in 2021, China’s industrial sulfur dioxide emissions reached 2.748 million tons，accounting for 76.31% of the total emissions. Excessive emissions of industrial pollutants have not only resulted in severe pollution of water, air, and soil, but also contributed to human health issues such as respiratory ailments, cardiovascular diseases, and blood disorders (4).

Currently, new technologies represented by industrial robots play a significant role in production, fundamentally altering production. These technologies also bring important green environmental benefits, making them one of the key channels for achieving pollution reduction (5). However, existing literature does not directly address the relationship between the adoption of robots and urban green development. On one hand, according to the research by Gutierrez and Teshima (6), production technology and emission reduction technology are the main channels for enterprises to reduce emissions. The adoption of robots leads to advancements in production technology, improving productivity (7) and reducing energy intensity, known as the production effect. Furthermore, advancements in production technology and scale expansion increase corporate investment in emission reduction equipment, improving emission reduction technology (8), known as the emission reduction effect. Both the production effect and the emission reduction effect contribute to reducing emission intensity. On the other hand, scale expansion increases the total amount of pollution emissions, known as the scale effect.

Although existing research has drawn some important conclusions regarding the impact of robots on public health, significant gaps remain. According to the data from National Safety Council (NSC), in 2022, the number of preventable work deaths reached 4,695, and work-related medically consulted injuries totaled 4.53 million, costing 167 billion dollars. Robot adoption can significantly improve the working environment and reduce high-risk and high-pollution factors in the production field. Specifically, a one standard deviation increase in robot adoption (1.34 robots per 1,000 workers) reduces work-related injury rates by approximately 1.2 injuries per 100 full-time workers (9). Prior research in public health has mainly focused on specific groups, including the older adult or migrant populations. Additionally, the indicators used to measure public health outcomes vary widely and are often relatively singular, lacking a comprehensive approach. Finally, few studies have discussed the integration of green industrial practices within urban production spaces in relation to public safety and health. This poses our prime research question: how does robot adoption contribute to the green development of urban industrialization, and what is its impact on public health?

**Figure 1:**
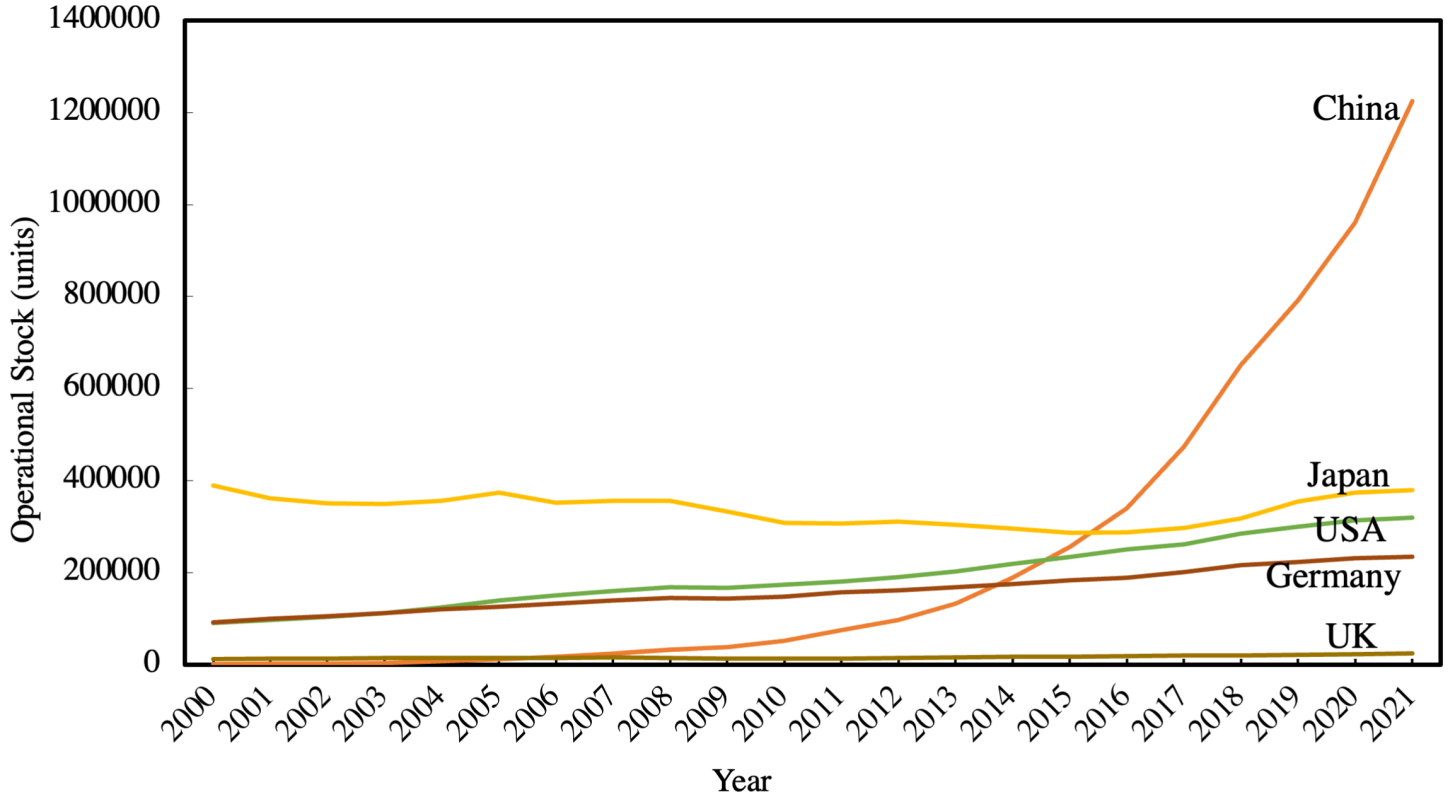
Number of operational robots, by Country, 2000–2021. Source: International Federation of Robotics (IFR) database.

To answer this question, China is a natural choice for a study of robotics because of its incredible jump from negligible numbers of robots in the early 2000s to the world’s leading user of industrial robots in the 2010s, deploying over half the world’s industrial robots as of 2021^1^ (see Figure 1). In recent years, China has been committed to reducing emissions and pollution, achieving significant results. From 2018 to 2021, China’s total investment in environmental pollution control has increased from 891.15 billion yuan to 949.18 billion yuan, industrial sulfur dioxide emissions have decreased from 5.161 million tons to 2.748 million tons, and industrial solid waste production also has decreased from 4152.69 million tons to 3970.06 million tons.^2^ Thus, it is crucial to thoroughly investigate the green innovation factors in production that affect the public health of urban residents.

This study utilizes both macro and micro data to validate the relationship between green production in urban areas and public health across different regions of China. The analysis is based on large-scale population data from the China Labor-force Dynamic Survey (CLDS). Various public health variables were employed, including self-rated health, illness status, and mental health indicators. The findings indicate that robot adoption in workplaces benefits both public physical and mental health. Additionally, robots significantly reduce adverse weather conditions (PM 2.5) and polluted water levels at the city level. At the micro level, robot adoption reduces pollution in work environments and enhances safety in production processes, contributing to improved urban green spaces and public health. By mitigating environmental hazards, the integration of robotic technology in industrial production promotes a healthier, more sustainable urban environment, enhancing the well-being of city residents.

Furthermore, considering the significant bias in the impact of robot adoption on the labor market (10), the analysis also examined the varying effects on different demographic groups. The adoption of robots has notably improved high-pollution, high-risk production environments, significantly enhancing individual health in heavy physical labor occupations. Evidence shows that the adoption of robots has a more pronounced positive impact on the physical and mental health of non-insured manual female workers who are older, have lower education levels, and come from rural households. This indicates that technological application in production can significantly raise public health standards, thereby improving the overall well-being of workers.

The marginal contributions of this paper are: first, in terms of research perspective, this paper extends the impact of industrial robots on the job market from the level of jobs and wages to the production environment and public health, which is a useful addition to the existing literature and enriches the relevant studies on the socio-economic impacts of industrial robots. Second, in terms of research content, this paper uses regional macro-pollution data as the basic indicator of public environmental level, and uses micro-individual survey data to measure the working environment and work safety of workers, and on this basis identifies the potential mechanism of the robot adoption affecting workers’ health (physical and mental health). Third, in terms of policy implications, the findings of this study will serve as a valuable resource for urban planners and decision makers, contributing to the enhancement of public health among urban residents.

The remainder of the paper is organized as follows: Part II provides a more detailed literature review on public health; Part III describes the data variables and empirical model; Part IV presents the baseline results; Part V discusses the heterogeneity and potential mechanisms behind the impact of robot adoption on public health; and the conclusion summarizes the main findings and provides suggestions for future research.

## 2 Literature review

### 2.1 Urbanization, robotics and green workplace

The advent of Industry 4.0 has seen industrial robots become a cornerstone of modern manufacturing, significantly enhancing production efficiency and economic benefits. These robots contribute to operational cost savings, labor productivity improvements, and increased total factor productivity (11). Notably, while the primary intent of deploying industrial robots is economic, their adoption has inadvertently yielded substantial environmental benefits. These benefits include waste reduction, improved energy efficiency, and the facilitation of cleaner production processes (12), which collectively lower carbon intensity and promote sustainable development.

Industrial robots have catalyzed technological innovation within enterprises, fostering knowledge creation, learning capabilities, research and development (R&D), and talent investment (13, 14). This technological innovation simplifies green process innovations and reduces carbon intensity, demonstrating a potential alignment between economic growth and environmental sustainability (15). The optimization of production factors, driven by the integration of industrial robots, leads to a reconfiguration of production resources, enhancing energy efficiency and reducing carbon emissions (16). Consequently, industrial robots contribute to improving urban green spaces and public health.

The relationship between technological progress and pollution has been debated since the pioneering studies by Ehrlich and Holdren (17) and Simon (18). While some researches indicate that technological advancements can mitigate pollution (19–21), other studies present mixed results (22–24). The introduction of industrial robots provides environmental benefits such as reduced material losses, opportunities for digitized environmental monitoring, and enhanced environmental accounting systems. However, potential risks include increased energy intensity and electronic waste from proliferated production.

The impact of industrial robots on the environment is mediated through various mechanisms. Energy-efficiency effects typically reduce energy consumption, while rebound effects and scale effects can increase it. Many studies have explored these countervailing forces (25, 26). The relationship between energy consumption and air pollution is well-documented, with increased energy consumption being a primary cause of pollution and carbon dioxide emissions (27, 28).

Research on the robot adoption and their environmental impacts has yielded insightful findings. For instance, Chen et al. (29) used panel data from 72 countries and regions and found that robots decreased the ecological footprint through time-saving, green employment, and energy upgrading effects. Singhania and Saini (30) utilized carbon emission data from 21 countries spanning from 1990 to 2016, finding that the technological effects in developing countries demonstrate that R&D has played a role in mitigating environmental degradation. Xu et al. (31) used data from 279 prefecture-level cities in China between 2007 and 2016, discovering that trade liberalization has a suppressive effect on haze pollution through technological spillovers. Luan et al. (32) indicated that industrial robots improve productivity and energy efficiency, thus reducing greenhouse gas emissions. Moreover, Song et al. (33) and Zhu et al. (34) have shown that robot adoption reduces firms’ pollution intensity, especially in high-pollution industries.

Green technology innovation, initially introduced as “sustainable development” in the 1980s and later termed ‘eco-innovation’ in the 1990s, has become a focal point in reducing environmental pollution. Feng et al. (35) and Wang et al. (36) demonstrate that green technology innovation significantly mitigates pollution, using methods like the Spatial lag model and the Spatial Durbin model to analyze panel data across various Chinese cities and provinces. These innovations not only improve energy efficiency but also reduce emissions, aligning with broader sustainable development goals.

Despite these positive findings, existing research on the environmental impact of industrial robots is limited and fragmented. Most studies focus on either energy consumption or green technology innovation without integrating both aspects into a comprehensive analysis. Moreover, empirical evidence at the individual or city level is scarce, with most research conducted at the national level.

### 2.2 Technological advancements and public health

The intersection of technological advancement and public health presents a multifaceted landscape with significant implications for occupational safety, environmental sustainability, and healthcare delivery. Recent literature highlights both the potential benefits and limitations of integrating these technologies into various sectors.

Recent studies argue that AI and robots can reduce occupational injuries. For instance, robots can replace workers in hazardous environments, such as chemical and mining industries, thereby mitigating exposure to dangerous conditions (37, 38). Moreover, robots can enhance workplace safety by monitoring environments and issuing alerts when unsafe conditions arise (39). In rescue operations, robots can prevent secondary injuries to rescue workers, thereby safeguarding rescue teams (40).

However, the efficacy of robots in reducing occupational injuries is debated. Despite advancements in AI, more than 90% of jobs cannot be fully automated due to the limitations of current AI technology (41). Robots often lack the flexibility, comprehension, cognitive capacity, and decision-making skills that human workers possess, making effective human-computer interaction challenging (42, 43). Additionally, programming defects and reliability issues can lead to communication breakdowns and accidents, sometimes resulting in injuries or fatalities (37, 44, 45). The successful adoption of robots in reducing occupational injuries hinges on two critical premises: accurate and stable robot programming and well-trained operators (46). In developed countries, where AI technology is more advanced, robots can operate more reliably, and human-computer interactions are more effective (47). However, in developing countries, technology limitations and inadequate training for robot operators pose significant challenges (48, 49).

The role of robots in healthcare, especially in the context of public health emergencies like the COVID-19 pandemic, is also gaining attention. Robots have been deployed for disinfection, delivering medications and food, measuring vital signs, and assisting in border controls. During the 2015 Ebola outbreak, workshops highlighted the potential roles of robots in clinical care, logistics, and reconnaissance. In clinical care, robots are used for disease prevention, diagnosis, and patient management (40). The digitalization of healthcare offers significant potential for improving public health. AI can support community health workers and educators by providing information and decision-making support, thereby enhancing health outcomes, especially in underserved communities (50, 51). Also, the deployment of robots in public health settings extends beyond clinical care to include social support. For example, social robots can provide continued social interaction for isolated individuals, addressing the mental health challenges posed by prolonged quarantine.

In conclusion, the integration of advanced technologies into public health and occupational safety offers significant potential but also poses challenges that require careful consideration.

## 3 Data and methodology

### 3.1 Data

#### 3.1.1 Explanatory variable

This study primarily bases the construction of explanatory variables on data from the International Federation of Robotics (IFR) database. IFR conducts an annual survey of global robot manufacturers, compiling first-hand data provided by these manufacturers to form the world robot statistics. Currently, the IFR database covers annual average installations and stock of industrial robots in over 100 countries and regions since 1993. It serves as an authoritative and comprehensive robot statistical database, widely used in relevant literature both domestically and internationally.

Following Acemoglu and Restrepo (7), we combine this data with the employment of various industries to construct an indicator of industrial robot adoption at city level. This indicator serves to measure the extent of industrial robot adoption (See details of indicator constructions in Appendix D). The equation for constructing the industrial robot adoption in this paper is as follows:


(1) $$\mathrm{robo}t_{ct}=\underset{s\in S}{\sum }l_{cs}^{2006}\frac{PR_{st}}{L_{s}^{2006}}$$

In Equation 1, $PR_{st}$ represents the stock of robots in industry s in China for year t . $L_{s}^{2006}$ denotes the number of employees in industry s in China in 2006, and $l_{cs}^{2006}$ indicates the number of employees in industry s in city c in 2006. Given the typical facts that China entered a period of rapid growth in robot adoption in 2006, we choose this year as the base period. This selection helps to eliminate the effects of industry employment fluctuations on the robot adoption, thereby enhancing the precision of the results.

#### 3.1.2 Dependent variable

This study primarily bases the construction of dependent variables on data from the China Labor-force Dynamics Survey (CLDS) database. It tracks urban and rural residents across China, creating a comprehensive database that includes longitudinal and cross-sectional data on labor force individuals, families, and communities. This database provides high-quality foundational data for empirical research and policy analysis. The survey is characterized by its wide range of topics and diverse representational levels, focusing on the current situation and changes in education, employment, labor rights, occupational mobility, occupational protection and health, job satisfaction, and well-being of the working-age population aged 15 to 64. It also covers samples from 29 provinces and cities across China, ensuring national representation, as well as representativeness in the eastern, central, and western regions, Guangdong Province, and the Pearl River Delta. Overall, the survey encompasses multiple topics related to the political, economic, and social development of the communities where the labor force resides, as well as the demographic structure, family wealth and income, family consumption, family donations, rural family production, and land of the labor force’s families.

We utilize questions from the CLDS micro-survey. With regard to the health of workers, we focuses on measuring both physical and mental health. Physical health refers to a good condition that enables a person to work normally without any hidden health risks, and directly affects the attendance rate, work efficiency and safety of workers. Mental health means that all aspects of the mind and the process of activity are in a good or normal state, and are related to the motivation, teamwork, innovation and job satisfaction of the workers. Thus, we also select variables to depict the physical health from two dimensions: self-rated health and illness status, and mental health constructed by a depression scale calculating the arithmetic mean. Also, we measure the quality of environments through the responses to the question about occupational safety and workplace pollution control.

#### 3.1.3 Control variable

We select a series of demographic characteristics as control variables to avoid potential estimation errors. At the micro level, the selection of control variables in this study is primarily based on demographic information from CLDS. The average age of workers in the sample is 52.7 years, with males accounting for 47.5% and rural household registrations accounting for 83.4%. At the macro level, control variables are constructed mainly based on data from the China Statistical Yearbook. GDP *per capita*, investment, average salary, and industrial structure are used to describe the economic status of the city, while number of physician is used to depict the level of medical care, and number of internet user is utilized to represent the level of technological advancement in the city. The statistical descriptions of the relevant indicators involved in this paper are shown in Table 1, and the detail of variable description is shown in Table A1 in Appendix A.

**Table 1:** Statistical description.

	Var	Obs.	Mean	Sta. Dev.	Min	Max
Dependent variable	Self-rated health	37,467	2.610	1.006	0	4
	Illness status	37,458	0.891	0.312	0	1
	Depression	37,515	1.372	0.464	0	4
	Medical care	4,083	0.378	0.485	0	1
	Hospitalization	37,458	0.910	0.287	0	1
	Occupational safety	30,197	2.581	0.842	0	4
	Pm2.5	3,739	44.453	19.631	3.382	110.121
	Industrial wastewater	3,658	7464.775	9456.857	7	91,260
	Workplace pollution control	30,254	2.472	0.870	0	4
Explanatory variable	Robot	31,630	1578.254	2547.638	0	20317.430
Control variable	Macro-level					
	GDP	3,703	2002.094	2739.727	51.9279	28178.65
	Investment	3,433	1228.512	1460.599	33.0703	17245.770
	Average salary	3,720	40117.090	17754.370	4,958	137085.500
	Industrial structure	3,747	44.24524	13.94521	1.8	84.4
	Number of physician	3,750	9385.709	8984.253	1	96,445
	Number of internet user	3,715	75.1086	138.2286	0.024	5,174
	Micro-level					
	Gender	37,515	0.475	0.499	0	1
	Age	37,515	52.663	14.537	11	104
	Household register	37,452	1.412	4.431	1	3
	Employment status	37,515	0.294	0.456	0	1
	Marital status	37,515	2.015	0.759	1	6
	Maladaptive behaviors	37,464	0.268	0.443	0	1

### 3.2 Identification strategy

This paper aims to explore the impact of robot adoption on public health. To achieve this, an empirical model is constructed as follows:


(2) $$\mathrm{healt}h_{ic}=\alpha_{3}+\beta_{3}\mathrm{robo}t_{c}+\gamma_{3}X_{ic}^{D}+\varepsilon_{ic}$$

Equation 2 is used to test the health-promoting effects of robot adoption. The subscript i represents the individual respondent, c represents the city, respectively. $\mathrm{healt}h_{ic}$ denotes the health status (i.e., self-rated health, illness status, depression) of respondent i , $\mathrm{robo}t_{c}$ indicates the robot adoption in the city c in the year t , $X_{ic}^{D}$ includes a series of individual characteristic variables (including, age, gender, household register, employment status, marital status, and maladaptive behaviors) and $\varepsilon_{ic}$ is an idiosyncratic error term.

To further explore the potential mechanisms behind the effects of robots on public health, we utilize both macro-and micro-level environmental variables. The regressions are as follows:


(3) $$\mathrm{regionenvironmen}t_{ct}=\alpha_{1}+\beta_{1}\mathrm{robo}t_{ct}+\gamma_{1}X_{ct}^{D}+\sigma_{c}+\theta_{t}+\varepsilon_{ct}$$

(4) $$\mathrm{workplaceenvironmen}t_{ic}=\alpha_{2}+\beta_{2}\mathrm{robo}t_{c}+\gamma_{2}X_{ic}^{D}+\varepsilon_{ic}$$

Equation 3 is based on a two-way fixed effects model using macro-level panel data, designed to test the impact of industrial robot on environmental quality. The subscripts c and t respectively represent the city and year. $\mathrm{regionenvironmen}t_{ct}$ represents the regional environmental quality (i.e., PM2.5, industrial wastewater) at the macro level for city c in year t , $\mathrm{robo}t_{ct}$ indicates the robot adoption in city c during year t . $X_{ct}^{i}$ includes a series of city characteristic variables (i.e., GDP, investment, average salary, industrial structure, physician number, internet user number), and $\varepsilon_{ct}$ represents an idiosyncratic error term. The model in Equation 3 also contains city fixed effects $\sigma_{c}$ and year fixed effects $\theta_{t}$ to control for unobservable city-invariant and time-invariant differences across cities that may affect regional environment. The variables robot, PM2.5, industrial wastewater, GDP, investment, average salary, physician number, and internet user number are log-transformed. Furthermore, Equation 4 examines the impact of industrial robot on workplace environmental quality at the micro-level, $\mathrm{workplaceenvironmen}t_{ic}$ represents the quality of the workplace environment (i.e., occupational safety, workplace pollution control) of respondent i in city c at the micro-level. The other settings of the model are consistent with those in Equation 2.

## 4 Main results

### 4.1 The impact of robot adoption on public health

In this section, we will explore the relationship between robot adoption and public health. With the continuous embedding of industrial robots in production field, China’s traditional production has been subjected to the corresponding impact and innovation in the process of robot adoption, which is reflected in the significant changes in the working environment and the content of the labourers’ work. Robots can undertake more physically demanding and high-pollution production tasks, thereby reducing the likelihood of worker injuries (52). However, the rapid advances in automation may also have a negative impact on the labor force participation, leading to a deterioration in the physical and mental health of the worker (53). Here, we base on several questions from CLDS to depict the physical health from two dimensions: self-rated health and illness status, and mental health constructed by a depression scale calculating the arithmetic mean.

Table 2 reports the impact of robot adoption on workers’ health status. The robot adoption has a significant positive correlation with public health, especially when control variables are included in the regression model. Specifically, for each unit increase in robot adoption in the city, self-rated health score increases by 0.048 units, injury and illness score increases by 0.03 units, and depression scale score decreases by 0.026 units. This indicates that robot adoption have significant positive effects on workers’ health across multiple dimensions. Overall, as industrial robots become increasingly embedded in the production domain, there is a continuous shift away from high-risk and high-pollution jobs through “automation of tasks” (54, 55). On one hand, this is conducive to directly improving workers’ physical health levels and reducing the incidence of occupational accidents and diseases. On the other hand, it also helps alleviate psychological stress among workers by making work tasks more relaxed and flexible and production environments safer and more controllable.

**Table 2:** The impact of robot adoption on public health.

	(1)	(2)	(3)	(4)	(5)	(6)
	Self-rated health		Illness status		Depression	
*robot*	0.054^***^	0.048^***^	0.002	0.003^**^	−0.023^***^	−0.026^***^
	(0.004)	(0.004)	(0.001)	(0.001)	(0.002)	(0.002)
_cons	2.260^***^	3.452^***^	0.876^***^	0.948^***^	1.512^***^	1.454^***^
	(0.026)	(0.033)	(0.008)	(0.011)	(0.012)	(0.016)
Control	N	Y	N	Y	N	Y
Observations	31,072	30,748	31,067	30,743	31,072	30,748
R-squared	0.117	0.118	0.009	0.009	0.018	0.019

(1) Standard errors are clustered at the individual level. (2) ^***^ Significant at the 1% level ^**^ Significant at the 5% level. ^*^ Significant at the 10% level.

### 4.2 Robustness check

In this section, we conduct several robustness checks. Firstly, taking into account the possible bias in the selection of indicators, we verify the robustness of the baseline regression by replacing with alternative dependent variables. Secondly, considering the dynamic adjustments in the labor market, where the impact of new technologies on labor may have a delayed response, and the gradual process of industrial robots from introduction to actual use and then to large-scale production, we also examine the lagged effects of industrial robot on the labor market as a part of our robustness checks.

#### 4.2.1 Alternative dependent variable

**Figure 2:**
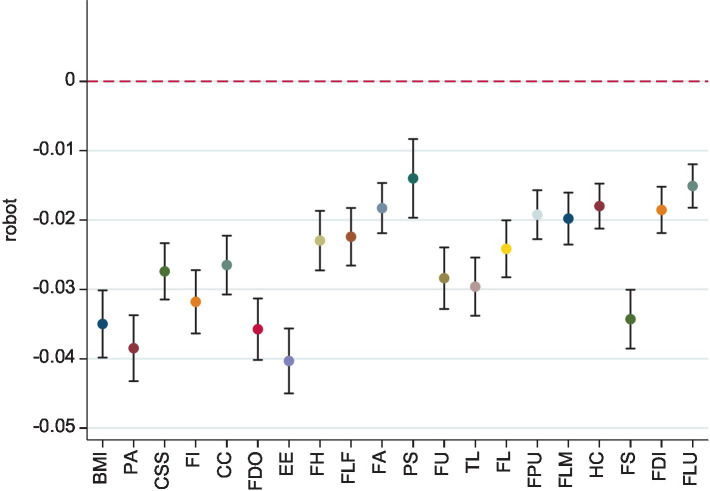
Robustness check on mental health. The regression model is referenced to Equation 4. The vertical line indicates the 90% confidence interval. See more details of regression tables in Appendix B. Specifically, BMI = bothered by minor issue, PA = poor appetite, CSS = cannot shake sadness even with help, FI = feel inferior, CC = cannot concentrate, FDO = feel down, EE = everything is an effort, FH = feel hopeless, FLF = feel life is a failure, FA = feel afraid, PS = poor sleep, FU = feel unhappy, TL = talk less, FL = feel lonely, FPU = feel people are unfriendly, FLM = feel life is meaningless, HC = have cried, FS = feel sorrowful, FDI = feel disliked, FLU = feel life is unlivable.

In Table 3, we replace the original dependent variables with medical care and hospitalization as alternative measures of worker health. The construction of the medical care and hospitalization indicators is based on questions from the CLDS questionnaire: “Have you sought medical attention due to illness or injury in the past 2 weeks?” and “In the past year, have you been diagnosed by a doctor as needing hospitalization?” respectively, with 0 representing “yes” and 1 representing “no.” Robot adoption still has positive effects on medical care and hospitalization. Additionally, in terms of workers’ mental health, we conducted separate regressions for the 20 questions that make up the depression scale, and the results are shown in Figure 2. These results further validate the positive impact of industrial robots on improving workers’ mental health.

**Table 3:** Robustness check on replacing dependent variable.

	(1)	(2)	(3)	(4)
	Medical care		Hospitalization	
*robot*	−0.001	0.001	0.008^***^	0.008^***^
	(0.005)	(0.006)	(0.001)	(0.001)
_cons	0.384^***^	0.421^***^	0.857^***^	0.987^***^
	(0.036)	(0.053)	(0.007)	(0.010)
Control	N	Y	N	Y
Observations	3,476	3,476	31,105	31,067
R-squared	0.001	0.010	0.002	0.020

(1) Standard errors are clustered at the individual level. (2) ^***^ Significant at the 1% level. ^**^ Significant at the 5% level. ^*^ Significant at the 10% level.

#### 4.2.2 Lagged effects

As shown in Table 4, we lag the robot adoption by one period and repeat the regression. The conclusions are consistent with the baseline regression, revealing that the health benefits of robot adoption have a certain degree of persistence. Besides, in terms of illness status and depression, the estimated coefficient of the lagged robot adoption rate is slightly larger than that of the current term, indicating that the positive effects of robot adoption on both physical and mental health have certain lags.

**Table 4:** Robustness check on lagged effect.

	(1)	(2)	(3)	(4)	(5)	(6)
	Self-rated health		Illness status		Depression	
*robot_(t-1)_*	0.052^***^	0.048^***^	0.003^**^	0.004^***^	−0.023^***^	−0.027^***^
	(0.004)	(0.004)	(0.001)	(0.001)	(0.002)	(0.002)
_cons	2.290^***^	3.472^***^	0.873^***^	0.946^***^	1.507^***^	1.444^***^
	(0.025)	(0.032)	(0.007)	(0.01)	(0.011)	(0.015)
Control	N	Y	N	Y	N	Y
Observations	30,787	30,748	30,779	30,743	30,830	30,748
R-squared	0.006	0.118	0.001	0.009	0.006	0.019

(1) Standard errors are clustered at the individual level. (2) ^***^ Significant at the 1% level. ^**^ Significant at the 5% level. ^*^ Significant at the 10% level.

### 4.3 Endogeneity

Although the baseline regression in the previous section controls for various individual characteristics, omitted variable bias may still pose endogeneity issues. Additionally, due to the common correlation between poor worker health and reduced work capacity, companies aiming to enhance productivity and maximize profits may have incentives to adjust the scale of “machine substitution” based on the health status of their workforce. This introduces reverse causality between robot adoption and worker health, further exacerbating endogeneity concerns.

In this section, we draw on the endogeneity handling method from Acemoglu and Restrepo (7). In their study on the employment effects of robot adoption in the United States, they used robot data from Germany, Japan, and South Korea—countries with rapid industrial robot adoption—to construct an instrumental variable for U.S. robot adoption rates. Similarly, we use the robot stock in the U.S. during the same period to construct an instrumental variable for China’s robot adoption rate. On one hand, with the increasing technological exchanges and cooperation between China and the U.S., the development and application trends of industrial robots in both countries exhibit a high degree of similarity, meeting the relevance requirement of an instrumental variable. On the other hand, the degree of robot adoption in the U.S. does not directly affect the health status of Chinese workers, satisfying the exogeneity requirement of an instrumental variable. The specific construction method of the instrumental variable is as follows:


(5) $$\mathrm{robo}t_{ct}^{\mathrm{US}}=\underset{s\in S}{\sum }l_{cs}^{2006}\frac{PR_{st}^{\mathrm{US}}}{L_{s}^{2006}}$$

In Equation 5, $PR_{st}^{\mathrm{US}}$ represents the robot stock in industry sss in the U.S. in year t , $L_{s}^{2006}$ represents the number of employees in industry s in China in 2006, and $l_{cs}^{2006}$ represents the number of employees in industry s in city c in China in 2006.

Table 5 reports the Two-Stage Least Squares (2SLS) estimations. In the first-stage regression, the coefficient of the U.S. robot adoption rate during the same period is significantly positive (*p* < 0.01), indicating a significant positive correlation between the U.S. and China robot adoption rates. The *F*-value is greater than 10, rejecting the weak instrument hypothesis. In the second-stage regression, the estimated coefficients of robot adoption rate for self-rated health, illness status, and depression are all significant (*p* < 0.01) and consistent with the baseline regression results.

**Table 5:** Result of IV regression.

	First-stage	Second-stage		
	robot	Self-rated health	Illness status	Depression
$\bar{\mathrm{robot}}$		0.121^***^	0.035^***^	−0.077^***^
		(0.026)	(0.009)	(0.012)
$\mathrm{robo}t^{\mathrm{US}}$	0.117^***^			
	(0.006)			
_cons	5.632^***^	3.045^***^	0.759^***^	1.757^***^
	(0.037)	(0.166)	(0.056)	(0.075)
Control	N	Y	Y	Y
*F-*statistics	711.785			
Observations	26,394	26,323	26,319	26,323

(1) Standard errors are clustered at the individual level. (2) ^***^ Significant at the 1% level. ^**^ Significant at the 5% level. ^*^ Significant at the 10% level.

### 4.4 Heterogeneity

Considering the significant bias in the impact of robot adoption on the labor market (10), and the fact that job content varies significantly across different occupational positions, as well as the presence of certain characteristic differences among worker groups, further heterogeneity analysis is needed to understand the effects of robot adoption on worker health.

The impact of robot adoption may vary among workers with different characteristics. Here, we use gender, age, household registration, and education level to describe the characteristics of worker groups. We use gender and age as the basis for categorizing worker groups to explore the impact on vulnerable groups in the labor market, such as female workers and middle-aged and older adult workers, in the context of robot adoption. For age group classification, we refer to the report published by the WHO, defining those aged 44 and below as young, those aged 45 to 59 as middle-aged, and those aged 60 and above as older adult. Additionally, human capital refers to the sum of economically valuable knowledge, skills, and physical abilities residing within workers, primarily reflected in their work ability, learning capability, and innovation skills (56). Thus, we use household registration and education level to characterize the human capital levels of workers, to explore the differences in impact experienced by worker groups with varying levels of human capital during the robot adoption. Here, we use educational level and type of household registration to characterize workers’ human capital levels. For household registration classification, we use the type of household registration at the time of the worker’s birth as the basis, dividing workers into two sample groups: rural household and urban household. For education classification, we define high school education and below as low education level, and associate degree, bachelor’s degree, and above as high education level.

Additionally, the impact of robot adoption may vary across different types of occupations. Here, we use job content and insurance status to characterize the features of these occupations. Cognitive labor primarily involves mental exertion, reflected in a worker’s scientific and cultural knowledge, production skills, and experience. In contrast, manual labor, or physical labor, primarily involves the musculoskeletal system (57). Due to differences in job content, different occupational positions are affected differently by the robot adoption. The CLDS questionnaire collects respondents’ occupational information and categorizes them into 16 occupational categories. Here, we define agriculture, forestry, animal husbandry, fishing, mining, manufacturing, and construction as heavy physical labor occupations, while finance and insurance, real estate, health care, sports and social welfare, education, culture and arts, broadcasting, film and television, scientific research, and comprehensive technical services, as well as electricity, gas, and water production and supply are defined as heavy cognitive labor occupations. And we classify insurance status based on participation in basic employee medical insurance.

**Figure 3:**
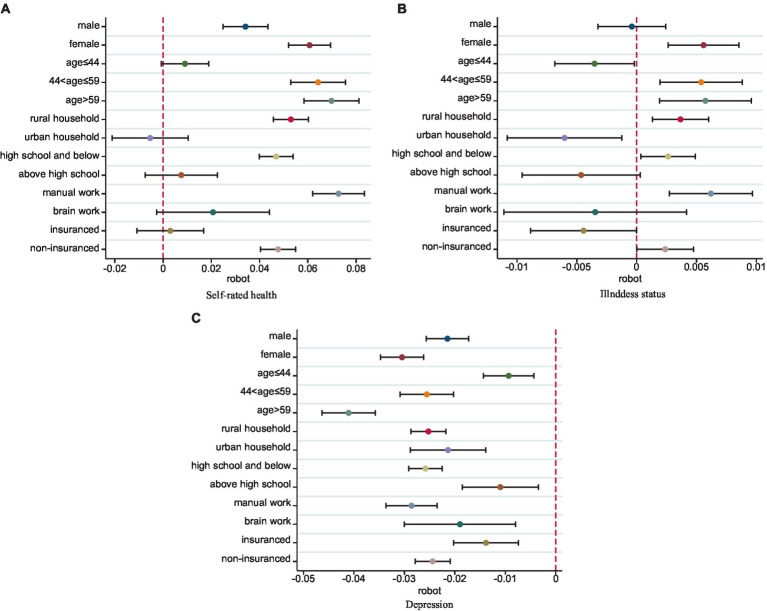
Heterogeneity. **(A–C)** are the results of the regression coefficients for self-rated health, illness status and depression as the dependent variable. Respectively, the regression model is referenced to Equation 4. The horizontal line indicates the 90% confidence interval. See details of regression tables in Appendix C.

In Figure 3, among different groups of workers, it is evident that the health improvement effects of robots are more significant in the vulnerable groups in the labor market. This inequality in effects is mainly evident in the dimensions of self-rated health and physiological health, indicating that female workers, middle-aged and older adult workers, and workers with low human capital benefit more from the health dividends brought by robot adoption. The reason may be that the health-promoting effects of industrial robots exhibit diminishing marginal returns. Vulnerable groups in the labor market are at a disadvantage in the labor market competition, leading them to be more concentrated in high-risk, high-pollution jobs. Compared to other workers, their working environments are worse, thus the improvements in their working conditions brought about by industrial robots are more pronounced (58).

Among different groups of occupations, it is observed that, in terms of self-rated health and physiological health, the health improvement effect of industrial robots is significant only among heavy physical labor and non-insuranced occupations. Regarding mental health, the regression coefficient of robot adoption is also higher among samples of workers in heavy physical labor occupations compared to those in heavy cognitive labor occupations. This could be attributed to the fact that robot adoption, as extensions of human limbs and senses, primarily manifest their health improvement effects through the replacement of high-risk and high-pollution jobs. At the same time, the lack of insurance often means that companies are unable to improve the safety of workers at work, which means that workers face more difficult working conditions. Hence, these effects are more pronounced in the domain of heavy physical labor and non-insuranced occupations.

## 5 Mechanism

In the previous section, we established the relationship between robot adoption and workers’ health. Next, we will explore the potential mechanisms at both macro and micro levels.

### 5.1 Environmental quality

Combining technological advancements with green transformation strategies can significantly reduce the opportunity cost of taking proactive climate actions while yielding substantial benefits (59). Specifically, robot adoption can promote capital-biased technological progress in enterprises. Increased investment in research and development not only enhances production efficiency and capacity but also improves enterprises’ technical capabilities in pollution reduction through technological spillovers (60). Thus, we use PM2.5 and industrial wastewater emissions to characterize the environmental quality of cities.

According to Table 6, robot adoption has a significant positive effect on environmental quality. Specifically, after controlling for various city characteristics, a 1% increase in robot adoption leads to a 0.9% decrease in city PM2.5 concentration and a 1.6% reduction in industrial wastewater discharge. This indicates that industrial robots can enhance workers’ health by improving environmental quality.

**Table 6:** The impact of robot adoption on environmental quality.

	(1)	(2)	(3)	(4)
	PM2.5		Industrial wastewater	
robot	−0.007^**^	−0.009^***^	−0.019^**^	−0.016^*^
	(0.003)	(0.003)	(0.008)	(0.009)
_cons	3.707^***^	6.472^***^	8.460^***^	6.828^***^
	(0.011)	(0.642)	(0.033)	(2.487)
Control	N	Y	N	Y
City fixed effect	Y	Y	Y	Y
Year fixed effect	Y	Y	Y	Y
Observations	3,739	3,346	3,658	3,337
R-squared	0.944	0.942	0.891	0.894

(1) Standard errors are clustered at the city level. (2) ^***^ Significant at the 1% level. ^**^ Significant at the 5% level. ^*^ Significant at the 10% level.

### 5.2 Workplace conditions

Industrial robots can handle tasks involving toxic substances or work in high-temperature and high-noise environments, reducing workers’ exposure to harmful materials and hazardous conditions, thereby lowering the risks of occupational diseases and injuries. Besides, Industrial robots undertake high-risk tasks like heavy lifting and operation in hazardous areas, improving overall workplace safety and reducing the occurrence of workplace accidents. Here, we use questions from the CLDS micro-survey such as “Does safety protection at work meet national standards?” and “Is workplace environmental pollution exceeding standards?” to measure the quality of workers’ micro work environments. This dual-level approach further investigates the role of workplace conditions in the health promotion effects of robot adoption.

According to Table 7, robot adoption also significantly improves workplace conditions. Specifically, after controlling for various city characteristics, for every 1 unit increase in robot adoption, the workplace pollution control score and occupation safety score increase by 0.012 and 0.019 units, respectively. This indicates that industrial robots can enhance workers’ health by improving workplace conditions.

**Table 7:** The impact of robot adoption on workplace conditions.

	(1)	(2)	(3)	(4)
	Workplace pollution control		Occupational safety	
robot	0.015^***^	0.012^***^	0.018^***^	0.019^***^
	(0.004)	(0.004)	(0.003)	(0.004)
_cons	2.374^***^	2.218^***^	2.474^***^	2.283^***^
	(0.024)	(0.037)	(0.023)	(0.036)
Control	N	Y	N	Y
Observations	25,425	25,378	25,378	25,331
R-squared	0.001	0.006	0.001	0.008

(1) Standard errors are clustered at the individual level. (2) ^***^ Significant at the 1% level. ^**^ Significant at the 5% level. ^*^ Significant at the 10% level.

## 6 Conclusion

In this paper we presented robust evidence of a highly significant relationship between public health and robot adoption, and analyze the role of workplace environmental quality in this relationship. Using the database from CLDS, we find robot adoption has a positive correlation with workers’ physical and mental health status. This conclusion remains robost after robustness tests and endogeneity issues are concerned. Additionally, taking into account the potential heterogeneity, we conduct group regression analysis on the sample based on workers’ and occupations’ characteristics, and find that manual and non-insuranced laborers and vulnerable groups in the labor market tend to enjoy more health benefits from robot adoption. Finally, we combine macro and micro data and find that robot adoption can achieve a positive impact on the health status of workers by improving environmental quality and workplace conditions.

With the continuous advancement of technologies represented by industrial robots, the urbanization process in developing countries is accelerating, and the large-scale trend of “machine substitution for human labor” in production is irreversible. While the impact of robots on workplace environmental quality and workers’ health is becoming increasingly profound, academic discussion in this field remains somewhat sparse. This paper aims to integrate relevant macro and micro data to provide empirical support from China’s experience in this field and offer academic evidence for pollution reduction and the improvement of workers’ health in China. Based on the results in this paper, the government should actively promote the widespread robot adoption in various sectors such as manufacturing, logistics, and construction. To achieve this, a series of corresponding policies should be implemented, including not only encouragement and support for research and innovation but also financial subsidies, tax incentives, and technical training. These measures can effectively foster the rapid development and widespread adoption of industrial robot, thereby fully leverage the potential of industrial robots to improve workplace environmental quality and workers’ health.

There are some limitations in this paper. Further refinement is possible in the characterization of environmental quality and worker health. In addition, the government significantly impacts workspaces and urban health through the protection of workers’ rights, the implementation of favorable technology policies, and environmental regulations. The specific direction and extent of this impact also warrant further discussion.

## Data availability statement

Publicly available datasets were analyzed in this study. This data can be found at: http://www.cnsda.org/index.php?r=projects/view&id=75023529 (China Labor-force Dynamics Survey).

## Author contributions

SY: Data curation, Investigation, Methodology, Project administration, Resources, Software, Validation, Writing – original draft, Writing – review & editing. MH: Investigation, Methodology, Writing – original draft, Writing – review & editing. JL: Data curation, Formal analysis, Software, Writing – original draft, Writing – review & editing. XY: Data curation, Software, Supervision, Writing – original draft, Writing – review & editing, Funding acquisition.

## Footnotes

^1^In 2021, the number of robot stock in China was 1,224,236 units.

^2^Source: China Environmental Statistical Yearbook.
