# Supplementary material for: Green workspace and urban health: exploring the impacts of industrial robotics in pollution emissions and public health
Source: Front Public Health. 2024 Aug 2;12:1445746. doi: 10.3389/fpubh.2024.1445746 (PMC11327072; doi:10.3389/fpubh.2024.1445746)
Supplement: Supplementary file 1 [file Table_1.docx]

**Appendix A**

Table A1 Variable description

| Variable | Description |
| --- | --- |
| self-rated health | “how do you assess your current health status?” 0=very unhealthy, 1=somewhat unhealthy, 2=average, 3=healthy, 4=very healthy |
| illness status | “have you been ill or injured in the past two weeks?” 0=yes, 1=no |
| depression | the frequency of “troubled by minor issues”, “loss of appetite, not feeling like eating”, “unable to shake off feelings of depression despite having family and friends’ support”, “feeling inferior to most people  difficulty concentrating on tasks”, “feeling emotionally low”, “feeling like everything is an effort”, “feeling hopeless about the future”, “feeling that life is a failure”, “feeling afraid”, “poor sleep”, “feeling unhappy”  speaking less than usual”, “feeling lonely”, “feeling people are not very friendly towards oneself”, “feeling life is meaningless”, “have cried”, “feeling sorrowful”, “feeling people don’t like oneself”, “feeling life cannot go on”, 1=never, 2=seldom, 3=sometimes, 4=always |
| robot | - |
| GDP per capita | - |
| investment (hundred million) | total investment in urban fixed assets |
| average salary (yuan) | average salary of staff |
| industrial structure (%) | percentage of secondary industry |
| physician number | - |
| internet user number (ten thousand) | - |
| gender | 0=female, 1=male |
| age | - |
| household register | 1=rural household, 2=urban household, 3=residential household |
| employment status | “have you worked since last year?” 0=no, 1=yes |
| marital status | 1=single, 2=first marriage, 3=remarriage, 4=divorced, 5=widowed, 6=cohabiting |
| maladaptive behaviors | “history of alcohol consumption” , 0=no, 1=yes |
| occupational safety | “satisfaction with safety protection at work” , 1=very unsatisfied, 2=unsatisfied, 3=average, 4=satisfied, 5=very satisfied |
| workplace pollution | “satisfaction with the work environment” , 1=very unsatisfied, 2=unsatisfied, 3=average, 4=satisfied, 5=very satisfied |
| PM2.5 | - |

**Appendix B**

**Table B1 Heterogeneity in work content**

|  | (1) | (2) | (3) | (4) | (5) | (6) |
| --- | --- | --- | --- | --- | --- | --- |
|  | self-rated health | | illness status | | depression | |
|  | manual work | brain work | manual work | brain work | manual work | brain work |
| $robot$ | 0.073*** | -0.021 | 0.006*** | -0.003 | -0.029*** | -0.019*** |
|  | (0.007) | (0.014) | (0.002) | (0.005) | (0.003) | (0.007) |
| _cons | 3.146*** | 3.438*** | 0.926*** | 0.907*** | 1.465*** | 1.496*** |
|  | (0.061) | (0.124) | (0.02) | (0.044) | (0.028) | (0.059) |
| Control | Y | Y | Y | Y | Y | Y |
| Observations | 12927 | 1901 | 12925 | 1901 | 12927 | 1901 |
| R-squared | 0.085 | 0.042 | 0.013 | 0.005 | 0.028 | 0.014 |

*Notes:* (1) Standard errors are clustered at the individual level. (2) *** Significant at the 1% level. ** Significant at the 5% level. * Significant at the 10% level.

**Table B2 Heterogeneity in education**

|  | (1) | (2) | (3) | (4) | (5) | (6) |
| --- | --- | --- | --- | --- | --- | --- |
|  | self-rated health | | illness status | | depression | |
|  | high school and below | above high school | high school and below | above high school | high school and below | above high school |
| $robot$ | 0.047*** | 0.008 | 0.003* | -0.005 | -0.026*** | -0.011** |
|  | (0.004) | (0.009) | (0.001) | (0.003) | (0.002) | (0.005) |
| _cons | 3.43*** | 3.5*** | 0.945*** | 0.958*** | 1.459*** | 1.452*** |
|  | (0.036) | (0.076) | (0.012) | (0.024) | (0.017) | (0.039) |
| Control | Y | Y | Y | Y | Y | Y |
| Observations | 27048 | 4024 | 27044 | 4023 | 27048 | 4024 |
| R-squared | 0.106 | 0.046 | 0.009 | 0.003 | 0.018 | 0.009 |

*Notes:* (1) Standard errors are clustered at the individual level. (2) *** Significant at the 1% level. ** Significant at the 5% level. * Significant at the 10% level.

**Table B3 Heterogeneity in household register**

|  | (1) | (2) | (3) | (4) | (5) | (6) |
| --- | --- | --- | --- | --- | --- | --- |
|  | self-rated health | | illness status | | depression | |
|  | rural household | urban household | rural household | urban household | rural household | urban household |
| $robot$ | 0.053*** | -0.005 | 0.004** | -0.006** | -0.025*** | -0.021*** |
|  | (0.004) | (0.01) | (0.001) | (0.003) | (0.002) | (0.005) |
| Control | Y | Y | Y | Y | Y | Y |
| _cons | 3.412*** | 3.846*** | 0.944*** | 1.021*** | 1.435*** | 1.511*** |
|  | (0.038) | (0.081) | (0.012) | (0.023) | (0.018) | (0.04) |
| Observations | 25604 | 3774 | 25599 | 3774 | 25604 | 3774 |
| R-squared | 0.12 | 0.098 | 0.01 | 0.011 | 0.022 | 0.011 |

*Notes:* (1) Standard errors are clustered at the individual level. (2) *** Significant at the 1% level. ** Significant at the 5% level. * Significant at the 10% level.

**Appendix C**

The construction of the industrial robot exposure index in this article primarily draws on Acemoglu and Restrepo (2020). Specifically, their research employs a model that examines the competition between robots and human labor across various tasks in production, analyzing the impact of industrial robot applications on the U.S. labor market. The findings suggest that an increased use of industrial robots has a negative effect on wages and employment. This model accounts for variations in the share of tasks that can be performed by robots across different industries and allows for inter-regional trade among labor markets specialized in producing different products. The settings are as follows:

The entire economy is composed of multiple regions $c$, with the total output $Y_{c}$ of a region being the sum of outputs $Y_{ci}$Yci from various industries $i$, weighted by the coefficients $v_{i}$. The parameter $\sigma$>0 represents the elasticity of substitution between goods produced by different industries.

The total output of region ccc is given by:

$$\begin{aligned} Y_{c}=\left( \sum_{i\in I} v_{i}^{\frac{1}{\sigma}}Y_{ci}^{\frac{\sigma-1}{\sigma}} \right)^{\frac{\sigma}{\sigma-1}}.\#\left( 5 \right) \end{aligned}$$

The quantity of consumer goods produced by industry$i$ in region $c$ is given by:

$$\begin{aligned} X_{ci}=\alpha^{-\alpha}\left( 1-\alpha\right)^{-\left( 1-\alpha\right)}A_{ci}\left[ \min_{s\in\left[ 0,1 \right]} x_{ci}\left( s \right) \right]^{\alpha}K_{ci}^{1-\alpha},\#\left( 6 \right) \end{aligned}$$

In Formula (6), $K_{ci}$ represents the non-robot capital, 1−$\alpha$ denotes the proportion of this factor in production, and $A_{ci}$ represents the productivity of the industry. The distribution of the number of production tasks $x_{ci}\left( s \right)$ between robots and workers is as follows:

$$\begin{aligned} x_{ci}\left( s \right)=\left\{ \begin{aligned} \gamma_{M}M_{ci}\left( s \right)+\gamma_{L}L_{ci}\left( s \right), &if s\leq\theta_{i}, \\ \gamma_{L}L_{ci}\left( s \right), &if s>\theta_{i}, \end{aligned} \right.\#\left( 7 \right) \end{aligned}$$

In Formula (7), $\gamma_{L}$ and $\gamma_{M}$ represent the productivity of workers and robots, respectively, both greater than 0. $L_{ci}\left( s \right)$ and $M_{ci}\left( s \right)$ denote the number of workers and robots employed in task $s$ respectively. The range $\left[ 0,\theta_{i} \right]$ indicates the extent to which tasks can be automated using robots; as time progresses, an increasing number of tasks are automated.

In region $c$, the supply of labor provided by households satisfies the following utility function:

$$\frac{C_{c}^{1-\psi}-1}{1-\psi}-\frac{B}{1+\epsilon}L_{C}^{1+\epsilon}.$$

The utility function for households in region $c$ must satisfy the following budget constraint: $C_{C}\leq W_{C}L_{C}+\Pi_{C}$. Here, $C_{C}$ represents the total consumption by the household, $L_{C}$ represents the supply of labor, $\psi$ denotes the income elasticity of labor supply, and $\epsilon$ represents the reciprocal of the wage elasticity of labor supply. This utility function must adhere to the constraint where $W_{C}$ denotes the wage per worker, and $\Pi_{C}$ represents the household’s non-labor income.

The investment required for producing robots is represented by the final goods, denoted by IcI_cIc. The number of robots is given by:

$$\begin{aligned} M_{c}=D\left( 1+\eta\right)I_{C}^{\frac{1}{1+\eta}},\#\left( 8 \right) \end{aligned}$$

In Formula (8),The number of robots increases with investment, where $\eta>0$ indicates that the marginal productivity of robots exhibits diminishing returns as investment increases. Additionally, let $R_{c}^{M}$ represent the rental rate of robots; the price of the fixed supply of capital $K_{c}$ within region $c$ is denoted by $R_{c}^{K}$.Thus, the equilibrium outcome within a region is determined by the equilibrium prices $W_{c}$, $R_{c}^{M}$, and $R_{c}^{K}$ (representing the prices of labor, robots, and capital respectively) and the equilibrium quantities $C_{c}$, $Y_{c}$, $I_{c}$, $L_{c}$, $M_{c}$ (representing the quantities of household consumption, total output, investment, labor supply, and the number of robots) across all regions $c\in C$.

The aforementioned equilibrium outcomes are derived from the conditions of profit maximization by firms, utility maximization by households, and the market-clearing conditions for labor, robots, capital, and final goods. These conditions ensure that all markets are in balance with no excess supply or demand, allowing the economy to function efficiently within each region:

$$\begin{aligned} \sum_{i\in I} \int_{0}^{1} L_{ci}\left( s \right)ds=L_{c},\#\left( 9 \right) \end{aligned}$$

$$\begin{aligned} \sum_{i\in I} \int_{0}^{1} M_{ci}\left( s \right)ds=M_{c},\#\left( 10 \right) \end{aligned}$$

$$\begin{aligned} \sum_{i\in I} K_{ci}\left( s \right)=K_{c},\#\left( 11 \right) \end{aligned}$$

$$\begin{aligned} C_{c}=Y_{c}-I_{c}.\#\left（ 12 \right） \end{aligned}$$

Extending the baseline model to an open economic environment, it can be derived and demonstrated that in all regions $\pi_{c}=\pi_{0}$ and in all industries $\theta_{i}=0$. Under these conditions, the following results hold:

$$\begin{aligned} \frac{dln L_{c}}{dt}=\left[ -\bar{\zeta}^{disp}\phi+\bar{\zeta}^{prod}\phi\pi_{0}-\bar{\zeta}_{L}^{inc}\psi\right]\sum_{i\in I} l_{ci}\frac{d\theta_{i}}{1-\theta_{i}}\frac{\gamma_{L}}{\gamma_{M}}+\bar{\zeta}_{L}^{Y}\frac{dln Y}{dt}+\bar{\zeta}_{L}^{\Pi}\frac{dln \Pi}{dt}+\bar{\zeta}_{cL}^{price},\#\left（ 13 \right） \end{aligned}$$

$$\begin{aligned} \frac{dln W_{c}}{dt}=\left[ -\bar{\zeta}^{disp}\epsilon+\bar{\zeta}^{prod}\epsilon\pi_{0}+\bar{\zeta}_{W}^{inc}\psi\right]\sum_{i\in I} l_{ci}\frac{d\theta_{i}}{1-\theta_{i}}\frac{\gamma_{L}}{\gamma_{M}}+\bar{\zeta}_{W}^{Y}\frac{dln Y}{dt}+\bar{\zeta}_{L}^{\Pi}\frac{dln \Pi}{dt}+\bar{\zeta}_{cW}^{price}.\#\left（ 14 \right） \end{aligned}$$

The variable $\zeta$ encapsulates the impact of robots on local employment and wages. The presence of inter-regional trade implies that productivity enhancements and price changes in one region will be shared across other regions. Since changes in national income $\text{dln}Y$, changes in non-national labor income $\text{dln}\Pi$, and price changes $\zeta^{price}$ are not functions of robot exposure rates, it allows us to use observational data to construct the core explanatory variable, the robot exposure rate in region $c$:

$$\underset{i\in I}{\sum}l_{ci}\frac{d\theta_{i}}{1-\theta_{i}}\frac{\gamma_{L}}{\gamma_{M}}.$$

The robot exposure index can be constructed using a Bartik / Shift-Share approach, which involves the interaction between the baseline employment shares of each industry in the local labor market and the technological feasibility of adopting robots at the industry level. This involves first calculating the number of robots per unit of employment at the industry level (robot exposure), and then multiplying this figure by the employment levels of the corresponding industries in each region. The product of these calculations represents the robot exposure at the regional level in the United States:

$$\begin{aligned} {APR}_{i}=\frac{d\theta_{i}}{1-\theta_{i}}\frac{\gamma_{L}}{\gamma_{M}}=\frac{dM_{i}}{L_{i}}-\frac{dY_{i}}{Y_{i}}\frac{M_{i}}{L_{i}},\#\left（ 15 \right） \end{aligned}$$

$$\begin{aligned} \text{US exposure to robots}_{c}=\sum_{i\in I} I_{ci}\cdot{APR}_{i}.\#\left（ 16 \right） \end{aligned}$$

In Formula (15) and (16), $I_{ci}$ represents the labor employment share of industry $i$ in region $c$; ${APR}_{i}$denotes the robot utilization rate within industry $i$ in the United States.

Based on the foregoing analysis, Acemoglu and Restrepo (2020) developed the metric of industrial robot exposure rate to assess the extent of industrial robot deployment. Furthermore, this metric has been extensively utilized by numerous scholars within the field of industrial robot applications, and it is recognized for its substantial representativeness and authoritative value.

Following the same conceptual framework for constructing this metric, we combine this data with the employment of various industries to construct an index of industrial robot exposure at city level. This index serves to measure the extent of industrial robot adoption. The specific formula for constructing the industrial robot exposure index in this paper is as follows:

$$\begin{aligned} {robot}_{ct}=\sum_{s\in S} l_{cs}^{2006}\frac{{PR}_{st}}{L_{s}^{2006}}\#\left( 17 \right) \end{aligned}$$

In Formula (17), ${PR}_{st}$ represents the stock of robots in industry $s$ in China for year $t$. $L_{s}^{2006}$​ denotes the number of employees in industry $s$ in China in 2006, and $l_{cs}^{2006}$​ indicates the number of employees in industry $s$ in city $c$ in 2006. To avoid the impact of fluctuations in employment numbers across different years and industries on the construction of the industrial robot application indicator, and combining this with the typical facts that China entered a period of rapid growth in the use of robots in 2006, we choose the year 2006 as the base period. This selection helps to eliminate the effects of industry employment fluctuations on the application of industrial robots, thereby enhancing the precision of the results.
